# Cancer risk following onset of type 2 diabetes in New Zealanders with impaired glucose tolerance over 25 years: a matched prospective cohort study

**DOI:** 10.1186/s12885-024-12637-4

**Published:** 2024-07-24

**Authors:** Zheng Wang, Dahai Yu, Uchechukwu Levi Osuagwu, Karen Pickering, John Baker, Richard Cutfield, Yamei Cai, Brandon J. Orr-Walker, Gerhard Sundborn, Bingjie Qu, Zhanzheng Zhao, David Simmons

**Affiliations:** 1 Department of Nephrology, the First Affiliated Hospital, Zhengzhou University, Zhengzhou, 450052 China; 2 Henan Institute of Interconnected Intelligent Health Management, First Affiliated Hospital, Zhengzhou University, Jianshe Road, Zhengzhou, Henan 450052 China; 3 Primary Care Centre Versus Arthritis, School of Medicine, Keele University, Keele, Staffordshire ST5 5BG UK; 4 Translational Health Research Institute (THRI), Western Sydney University, Campbelltown, Sydney NSW 2560 Australia; 5 School of Medicine, Western Sydney University, Campbelltown, Sydney NSW 2751 Australia; 6 Diabetes Foundation Aotearoa, Otara, New Zealand; 7 Department of Diabetes and Endocrinology, Counties Manukau Health, Auckland, New Zealand; 8 Section of Pacific Health, the University of Auckland, Auckland, New Zealand

**Keywords:** Impaired glucose tolerance, Type 2 diabetes, Cancer, Tapered matching, Landmark analysis, New Zealand

## Abstract

**Background:**

In people with prediabetes, the link between developing type 2 diabetes (T2D) and cancer risk among those with impaired glucose tolerance (IGT) remains uncertain. We examined this association in IGT individuals from primary care in South and West Auckland, New Zealand, spanning 1994–2019, assessing 5- and 10-year cancer risks.

**Methods:**

Study cohorts were extracted from the Diabetes Care Support Service in Auckland, New Zealand, linking it with national registries for death, cancer, hospital admissions, pharmaceutical claims, and socioeconomic status. We compared cancer risks in individuals with IGT newly diagnosed with or without T2D within a 1–5-year exposure window. Employing tapered matching and landmark analysis to address potential confounding effects, we formed comparative IGT cohorts. Weighted Cox regression models were then employed to assess the association between T2D onset and 5- and 10-year cancer risks.

**Results:**

The study included 26,794 patients with IGT, with 629 newly diagnosed with T2D within 5 years and 13,007 without such a diagnosis. Those progressing to T2D had similar 5-year cancer risk but significantly higher 10-year risk (HR 1.35; 95% CI 1.09–1.68). This association was stronger in older individuals, the socioeconomically deprived, current smokers, those with worse metabolic measures, and lower renal function. Patients with IGT of NZ European ethnicity had lower 10-year cancer risk.

**Conclusions:**

T2D diagnosis influences cancer risk in individuals with IGT. Developing risk scores for high-risk IGT individuals and implementing cancer screening and structured diabetes prevention, especially in deprived or minority ethnic populations, is essential.

**Supplementary Information:**

The online version contains supplementary material available at 10.1186/s12885-024-12637-4.

## Background

Cancer is a leading cause of death worldwide, accounting for nearly 10 million deaths in 2020 alone [1]. The burden of cancer is expected to rise in the coming years, with an estimated 28.4 million new cancer cases predicted by 2040 [1]. Type 2 diabetes (T2D) is also a significant public health concern globally, with an estimated 463 million people living with the condition in 2019 [2]. There is a growing body of evidence that suggests an increased risk of several types of cancer in individuals with T2D compared to the general population [3].

Impaired glucose tolerance (IGT) is a pre-diabetic state in which blood glucose levels are higher than normal but not high enough to be classified as T2D [4]. To date, several studies have examined the association between prediabetes or IGT and the risk of cancer [5] and the risk of cancer death [6–9] [10] with mixed results. A meta-analysis reported that individuals with prediabetes or IGT had a significantly higher risk of developing cancer compared to those with normal glucose levels, as IGT was associated with an overall 25% increased risk of cancer [11]. Recently In a Chinese general population, it was also found that individuals with IGT had a significantly increased risk of cancer [12].

However, no study has specifically investigated the association between the development of T2D and the risk of cancer in individuals with IGT, investigating the mediating effect of the onset of T2D in the association between IGT and cancer [13] [14]. Therefore, our study aims to fill this gap in the literature by using a novel landmark analysis with tapered matching strategies within a large IGT cohort to investigate the association between the onset of T2D and the risk of common adult cancers in New Zealand (NZ).

## Methods

### Data setting

The Diabetes Care Support Service (DCSS) was created in 1991 to improve diabetes care in West, East and South Auckland, NZ via general practice audits [18]. The DCSS also collected data on those with IGT. For this study, we identified a cohort of patients aged 18 years and above with IGT by linking the de-identified DCSS database with national cancer and death registration, hospitalization, pharmaceutical claim, and socioeconomic status data.

IGT was diagnosed using the 2-h glucose of 7.8–11 mmol/L on an oral glucose tolerance test (OGTT) [15]. The final dataset included demographics, clinical measurements (smoking, blood pressure (BP), body mass index (BMI), HbA1c, and lipids), and treatment (e.g., antihypertensive, statin, antiplatelet, and/or anticoagulant treatment). We validated the data through internal quality control policies and audits [16–18]. To cross-validate the prescription data in DCSS, we used pharmaceutical claims data from 2006 onwards as National Health Index numbers were not universal before then. We included data for all patients from their first DCSS enrolment date until their last enrolment on 31/7/2018. The North Health Ethics Committee approved the DCSS for research purposes in 1992, and then as an ongoing audit in 1996 (92/006). The ethics review was waived on March 25, 2019. We used anonymized data for this analysis, and signed consent was provided by an authorized signatory for each general practice. This manuscript adheres to the Strengthening the Reporting of Observational Studies in Epidemiology (STROBE) reporting guideline.

### Exposure

We identified patients with IGT and classified them based on exposure to T2D. Exposure was defined as newly diagnosed T2D recorded in any linked dataset. A landmark analysis was conducted to examine the effect of T2D onset on the risk of cancer. The analysis involved selecting a fixed time after cohort entry to conduct a survival analysis. Only patients with IGT who were alive at the landmark date were included, and T2D onset was based on exposure before the landmark date. Exposure was only evaluated during the exposure window, which was between the index date and the landmark time point. The outcome was then assessed from the landmark time point. Five landmark time points were determined a priori, specifically at 1, 2, 3, 4, and 5 years after the cohort enrolment date. Exposure status was assigned for patients with IGT who were still alive at the landmark dates. The method of landmark analysis was illustrated in Supplemental Fig. 1.

**Fig. 1:**
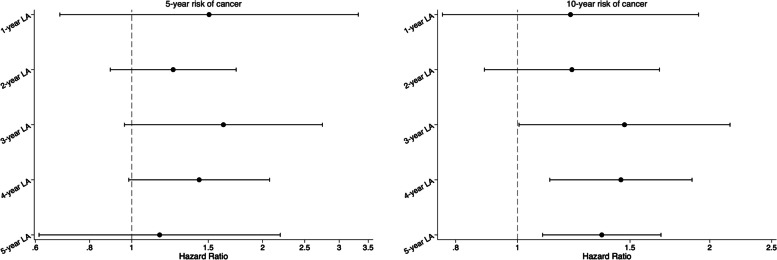
Adjusted hazard ratio for 5- and 10-year risk of cancer at 1-, 2-, 3-, 4-, and 5-year landmark (final tapered matched models)

### Outcome

The study assessed incident primary cancers as the outcome of interest. Incident cancer was defined as the first coded case of cancer recorded in the linked datasets, occurring during the follow-up period since the landmark time point, to mitigate potential information bias. Participants with IGT were followed up until an outcome of interest occurred or until December 31, 2019, for those without any outcome of interest. Outcomes were identified using primary International Classification of Diseases, Ninth Revision (ICD-9) and ICD-10 codes.

### Covariates

The potential confounding factors, including patient demographic characteristics (age, sex), lifestyle factors (smoking status), clinical measurements BMI, BP, HbA1c, lipids, eGFR), and treatments such as antihypertensive, anticoagulant, and lipid-lowering drugs at baseline, were considered as covariates in the analysis. The NZDep2013 Index of Deprivation, which provides an index of multiple deprivation (IMD) score for each meshblock in NZ based on the distribution of the first principal component scores, was used to define the socioeconomic status of participants [19]. The IMD score ranges from 1 to 10, with lower scores indicating less deprivation. To ensure sufficient statistical power, the IMD was categorized into five groups: IMD-1 (least deprived: NZDep2013 scores of 1–2), IMD-2, IMD-3, and IMD-4 (scores of 3–4, 5–6, and 7–8, respectively), and IMD-5 (most deprived: scores of 9–10). These categories were consistent with prior deprivation measures.

#### Statistical analysis

We employed tapered matching techniques to address confounding [20]. This approach evaluated the impact of T2D onset on the risks of cancers between focal (exposed: IGT with T2D onset during the exposure time window) and control (IGT without T2D onset during the exposure time window) groups using entropy balancing. This method involved gradually matching the control cohort to the focal cohorts using additional covariates and observing how the matched cohort changed with respect to hazard ratios (HRs) and unmatched covariates.

To minimize model dependence and the possibility of irresolvable imbalances between comparative groups, we used coarsened exact matching (CEM) to limit the comparison of patients in comparative groups to areas of common support before tapered matching and balancing [21, 22]. For each of the five years of landmark analysis, ten matching steps were performed, and patients with IGT in the comparative groups who were matched on the tenth step were retained (Supplemental Fig. 2–6).

**Fig. 2:**
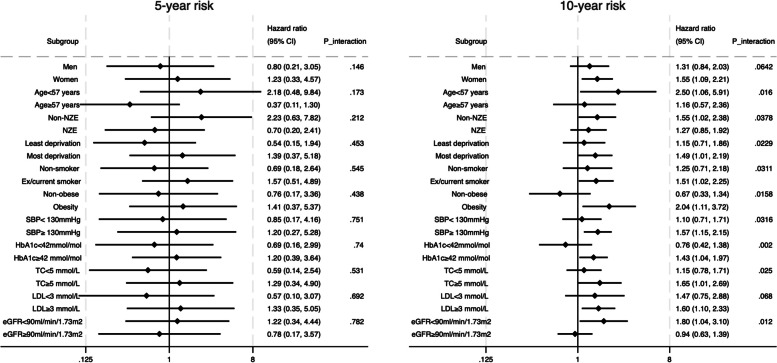
Stratified adjusted hazard ratio for 5- and 10-year risk of cancer at 5-year landmark (final tapered matched models**) **

We restricted the analysis to participants with areas of common support and used entropy balancing to minimize differences in matching variable distribution between comparison groups. Entropy balancing involves the maximum entropy reweighting of the unexposed group by directly incorporating covariate balance into the weight function, in which the matched sample is reweighted in each matching step to achieve key target moments such as mean, variance, and skewness. All pre-processing was conducted without reference to outcomes.

We applied weighted Cox proportional hazards regression, incorporating matching weights estimated from each matching step by entropy balancing, to account for competing risk of all-cause death (except deaths due to incident cancer). The analysis estimated the relative risk of cancer between comparison groups. Missing data were minimal, and multiple imputations with chained equations were performed on six imputed datasets using Robin's rule. Subgroup analysis was also processed by sex, age-group, NZE, deprivation status, smoking status, obesity, levels of clinical measurements (SBP, TC, LDL, and eGFR). Subgroup analyses employed a test of interaction to investigate whether there was evidence indicating a differential impact of T2D onset on the risk of cancer across subgroups. Analyses were conducted using Stata/MP version 17.0 (StataCorp LLC), and statistical significance was set at *P* < 0.05 (two-tailed).

## Results

A total of 26,794 patients with IGT were initially included in the study conducted by DCSS from 1994–2018. Participants with a history of outcomes, death, or loss of follow-up between the enrolment date and the landmark time point were excluded. Through 10 matching steps, matched cohorts of patients with and without the onset of T2D were created for 1-, 2-, 3-, 4-, and 5-year landmark analysis (Supplemental Fig. 2–6). The number of participants in the exposed vs. unexposed group were 112 vs. 1435, 254 vs. 2,872, 385 vs. 3,818, 477 vs. 3,922, and 511 vs. 3,336 controls, respectively.

Supplemental Table 1 and Table 1 display the characteristics of individuals with IGT with and without the onset of T2D before and after matching. After tapered matching, particularly entropy matching, no significant differences were detected in the variables included in the matching process between patients with IGT with and without the onset of T2D for all landmark analyses (Table 1), indicating successful matching.

**Table 1:** Comparison of patients with and without the onset of type 2 diabetes among patients with impaired glucose tolerance in the final entropy balancing matched cohorts

	**1-year landmark**			**2-year landmark**			**3-year landmark**			**4-year landmark**			**5-year landmark**		
	Without T2D onset	With T2D onset	*P*-value	Without T2D onset	With T2D onset	*P*-value	Without T2D onset	With T2D onset	*P*-value	Without T2D onset	With T2D onset	*P*-value	Without T2D onset	With T2D onset	*P*-value
	*N* = 21,648	*N* = 134		*N* = 21,283	*N* = 297		*N* = 19,520	*N* = 454		*N* = 16,681	*N* = 567		*N* = 13,007	*N* = 629	
No in the final matched cohort	*n* = 1,435	*N* = 112		*n* = 2,872	*n* = 254		*n* = 3,818	*n* = 385		*n* = 3,922	*n* = 477		*n* = 3,336	*n* = 511	
Age, years	55.8 (13.4)	56.5 (12.6)	0.280	55.8 (12.6)	57.5 (11.5)	0.112	55.2 (12.3)	56.7 (12.5)	0.123	54.9 (12.4)	55.9 (12.5)	0.221	55.2 (11.8)	55.6 (12.2)	0.548
Female Gender, % (SE)	60.0 (0.03)	60.2 (0.05)	0.9656	53.5 (0.02)	53.6 (0.04)	0.9743	51.4 (0.02)	51.5 (0.03)	0.9802	54.3 (0.02)	54.3 (0.03)	0.9819	54.6 (0.02)	54.6 (0.03)	0.9835
New Zealand European, % (SE)	37.3 (0.03)	37.3 (0.05)	0.9905	39.7 (0.02)	39.7 (0.04)	0.9932	38.3 (0.02)	38.3 (0.03)	0.9942	39.1 (0.02)	39.1 (0.03)	0.9952	39.0 (0.02)	39.1 (0.03)	0.9956
Enrol cohort, % (SE)															
1994–1998	1.1 (0.01)	2.4 (0.02)	0.2080	1.0 (0.01)	1.3 (0.01)	0.9520	0.9 (0.01)	0.9 (0.01)	1.0000	0.9 (0.01)	0.7 (0.01)	0.9831	1.2 (0.01)	0.6 (0.004)	0.4690
1999–2003	9.0 (0.03)	3.6 (0.02)		8.1 (0.02)	6.6 (0.02)		8.0 (0.02)	7.9 (0.02)		6.6 (0.01)	7.3 (0.02)		6.1 (0.01)	8.4 (0.02)	
2004–2008	9.3 (0.02)	16.9 (0.04)		14.7 (0.02)	16.6 (0.03)		15.1 (0.02)	15.0 (0.02)		15.4 (0.01)	14.2 (0.02)		18.7 (0.02)	15.0 (0.02)	
2009–2013	47.0 (0.03)	42.2 (0.05)		46.8 (0.02)	45.7 (0.04)		46.1 (0.02)	46.3 (0.03)		53.1 (0.02)	54.0 (0.03)		60.9 (0.02)	63.4 (0.03)	
2014–2018	33.6 (0.03)	34.9 (0.05)		29.4 (0.02)	29.8 (0.04)		29.9 (0.01)	30.0 (0.03)		24.0 (0.01)	23.9 (0.03)		13.1 (0.02)	12.5 (0.02)	
IMD group (NZDep13 scale), % (SE)															
Least deprivation: IMD-1 (1 or 2)	9.9 (0.02)	9.6 (0.03)	0.9967	6.6 (0.01)	6.0 (0.02)	0.7053	7.1 (0.01)	7.0 (0.02)	1.0000	7.7 (0.01)	8.0 (0.02)	0.9656	7.7 (0.01)	7.8 (0.02)	0.9944
IMD-2 (3 or 4)	9.1 (0.01)	9.6 (0.03)		14.3 (0.01)	16.6 (0.03)		16.7 (0.01)	16.7 (0.02)		16.3 (0.01)	15.2 (0.02)		15.9 (0.01)	15.3 (0.02)	
IMD-3 (5 or 6)	12.0 (0.03)	10.8 (0.03)		15.6 (0.02)	11.9 (0.03)		11.6 (0.01)	11.5 (0.02)		11.4 (0.01)	12.8 (0.02)		11.1 (0.01)	11.9 (0.02)	
IMD-4 (7 or 8)	19.2 (0.02)	20.5 (0.04)		15.1 (0.02)	17.9 (0.03)		15.1 (0.02)	15.4 (0.02)		14.9 (0.01)	14.2 (0.02)		15.7 (0.01)	15.3 (0.02)	
Most deprivation: IMD-5 (9 or 10)	49.7 (0.03)	49.4 (0.06)		48.4 (0.02)	47.7 (0.04)		49.4 (0.02)	49.3 (0.03)		49.6 (0.02)	49.8 (0.03)		50.0 (0.02)	49.7 (0.03)	
Smoking status, % (SE)															
Never smoking	55.8 (0.03)	55.4 (0.05)	0.9939	50.5 (0.02)	50.3 (0.04)	0.9972	53.8 (0.02)	53.7 (0.03)	0.9980	54.1 (0.02)	54.0 (0.03)	0.9984	53.5 (0.02)	53.4 (0.03)	0.9988
Ex-smoker	28.3 (0.03)	28.9 (0.05)		33.5 (0.02)	33.8 (0.04)		29.3 (0.02)	29.5 (0.03)		28.9 (0.01)	29.1 (0.03)		29.5 (0.02)	29.7 (0.03)	
Current Smoker	15.9 (0.02)	15.7 (0.04)		16.0 (0.01)	15.9 (0.03)		16.8 (0.01)	16.7 (0.02)		17.0 (0.01)	17.0 (0.02)		16.9 (0.01)	16.9 (0.02)	
Body mass index, kg/m^2^	33.8 (7.4)	33.8 (7.4)	0.953	33.4 (7.1)	33.5 (7.1)	0.961	33.9 (7.1)	33.9 (7.1)	0.970	33.5 (6.8)	33.6 (6.8)	0.972	33.9 (6.9)	33.9 (6.9)	0.975
Systolic blood pressure, mmHg	133 (17)	132 (17)	0.077	133 (16)	131 (17)	0.214	132 (16)	131 (17)	0.374	133 (16)	132 (17)	0.205	133 (17)	131 (16)	0.134
Diastolic blood pressure, mmHg	81 (10)	80 (11)	0.114	80 (10)	80 (11)	0.210	80 (10)	80 (11)	0.791	81 (10)	81 (11)	0.960	81 (10)	81 (10)	0.437
HbA1c, mmol/mol	43.3 (3.5)	43.3 (3.5)	0.879	43.8 (3.8)	43.8 (3.8)	0.911	44.5 (4.2)	44.5 (4.2)	0.938	44.9 (4.0)	44.9 (4.0)	0.942	45.0 (3.9)	45.1 (3.9)	0.945
Total cholesterol, mmol/L	4.9 (1.0)	4.9 (1.0)	0.949	4.8 (0.9)	4.8 (0.9)	0.956	4.8 (0.9)	4.8 (0.9)	0.966	4.8 (0.9)	4.8 (0.9)	0.969	4.8 (0.9)	4.8 (0.9)	0.974
Triglyceride, mmol/L	1.6 (0.7)	1.6 (0.7)	0.666	1.8 (0.8)	1.7 (0.7)	0.231	1.8 (0.8)	1.8 (0.8)	0.978	1.8 (0.8)	1.8 (0.8)	0.943	1.7 (0.8)	1.7 (0.8)	0.752
Low-density lipoprotein cholesterol, mmol/L	2.7 (0.7)	2.7 (0.7)	0.997	2.7 (0.7)	2.7 (0.7)	0.969	2.8 (0.7)	2.7 (0.7)	0.977	2.7 (0.8)	2.7 (0.8)	0.980	2.8 (0.7)	2.8 (0.7)	0.982
High-density lipoprotein cholesterol, mmol/L	1.3 (0.4)	1.3 (0.4)	0.239	1.3 (0.4)	1.3 (0.4)	0.496	1.3 (0.4)	1.3 (0.4)	0.907	1.2 (0.4)	1.2 (0.3)	0.888	1.2 (0.4)	1.2 (0.3)	0.271
estimated Glomerular filtration rate < 90 ml/min/1.73 m^2^	39.3 (0.02)	39.8 (0.05)	0.9966	37.5 (0.02)	37.7 (0.04)	0.9980	37.3 (0.02)	37.4 (0.03)	0.9986	42.4 (0.02)	42.6 (0.03)	0.9990	34.5 (0.02)	34.4 (0.03	0.9995
Antihypertensive treatment, % (SE)	26.5 (0.03)	26.5 (0.05)	0.9925	29.1 (0.02)	29.1 (0.04)	0.9948	31.7 (0.02)	31.7 (0.03)	0.9948	31.1 (0.02)	31.1 (0.03)	0.9955	35.9 (0.02)	35.9 (0.03)	0.9955
Statin treatment, % (SE)	24.0 (0.03)	24.1 (0.05)	0.9931	26.5 (0.02)	26.5 (0.04)	0.9952	28.2 (0.02)	28.2 (0.03)	0.9952	28.7 (0.02)	28.7 (0.03)	0.9958	34.0 (0.02)	34.1 (0.03)	0.9957
Antiplatelet or anticoagulant treatment, % (SE)	1.2 (0.01)	1.2 (0.01)	0.9984	0.7 (0.004)	0.7 (0.01)	0.9993	0.4 (0.002)	0.4 (0.004)	0.9994	0.3 (0.001)	0.3 (0.003)	0.9997	0.3 (0.002)	0.3 (0.003)	0.9997

N stands for the sample size for the cohorts before matching; n stands for the sample size for the cohort after matching. Continuous variables were presented as weighted means (standard error). Categorical variables were presented as weighted prevalence (standard error). T2D indicates type 2 diabetes

Table 2 presents the results of the landmark analyses, which show that the 5-year risk of cancer decreased over time for both the exposure and non-exposure groups. In the exposure group, the 5-year risk decreased from 22.92 (95% confidence interval: 11.44–41.00) per 1,000 person-years at the 1-year landmark analysis to 3.19 (1.38–6.29) at the 5-year landmark analysis. Similarly, in the non-exposure group, the 5-year risk decreased from 10.55 (8.10–13.49) to 2.56 (1.84–3.46) over the same period.

**Table 2:** 5-year and 10-year rates of any cancer among patients compared between people with impaired glucose tolerance with and without the onset of type 2 diabetes after coarsened and exact matching for 1-5 year landmark analysis

	**5-year risk**		**10-year risk**	
	**Exposure: with onset of T2D**	**Non-exposure: without onset of T2D**	**Exposure: with onset of T2D**	**Non-exposure: without onset of T2D**
	**Rate (95% CI), per 1,000 person-years**	**Rate (95% CI), per 1,000 person-years**	**Rate (95% CI), per 1,000 person-years**	**Rate (95% CI), per 1,000 person-years**
**1-year landmark analysis**	22.92 (11.44–41.00)	10.55 (8.10–13.49)	21.24 (11.31–36.32)	11.51 (9.16–14.26)
**2-year landmark analysis**	16.40 (9.72–25.93)	9.10 (7.47–10.98)	17.34 (11.22–25.59)	10.07 (8.50–11.84)
**3-year landmark analysis**	12.31 (7.62–18.82)	5.50 (4.42–6.76)	13.18 (8.89–18.81)	7.02 (5.90–8.29)
**4-year landmark analysis**	6.28 (3.44–10.54)	3.57 (2.76–4.56)	8.57 (5.60–12.56)	5.35 (4.44–6.39)
**5-year landmark analysis**	3.19 (1.38–6.29)	2.56 (1.84–3.46)	7.51 (4.95–10.91)	4.46 (3.61–5.44)

CI indicates confidence interval. T2D indicates type 2 diabetes

For the 10-year risk of cancer, the results were similar, with both the exposure and non-exposure groups showing a decreasing risk over time. Specifically, the 10-year risk in the exposure group decreased from 21.24 (11.31–36.32) per 1,000 person-years at the 1-year landmark analysis to 7.51 (4.95–10.91) at the 5-year landmark analysis. In the non-exposure group, the 10-year risk decreased from 11.51 (9.16–14.26) to 4.46 (3.61–5.44) over the same period.

After final step-10 matching, the final adjusted hazard ratios (HRs) for the 5-year risk of cancer comparing individuals with and without the onset of T2D decreased over the length of the landmark periods from 1.51 (0.68–3.32) at the 1-year landmark analysis to 1.16 (0.61–2.20) at the 5-year landmark analysis (see Fig. 1 and Supplemental Fig. 7). For the 10-year risk of cancer, the final adjusted HRs comparing individuals with and without the onset of T2D increased over the length of the landmark periods from 1.21 (0.76–1.92) at the 1-year landmark analysis to 1.35 (1.09–1.68) at the 5-year landmark analysis (see Fig. 1 and Supplemental Fig. 8).

In the stratified 5-year landmark analysis, the significant association between onset of T2D and risk of cancer was not found (see Fig. 2). For the 10-year cancer risk, the adjusted HR was significantly higher patients aged less than 57 years, those with non-NZE ethnicity, the most deprived, smokers, obese individuals, those with higher SBP and HbA1c levels, higher TC, and lower eGFR (see Fig. 2).

## Discussion

The present study investigated the association between the development of T2D within 1 to 5 years and the 5- and 10-year risk of cancer using landmark analysis in a population with impaired glucose tolerance in Auckland, NZ. The results showed increased final adjusted hazard ratios (HRs) for the 10-year risk of cancer comparing individuals with and without the onset of T2D increased from 1.21 at the 1-year landmark analysis to 1.35 at the 5-year landmark analysis. Our stratified analysis further highlights the importance of cancer prevention measures in the IGT population, such as smoking cessation, lifestyle modification, and screening strategies, especially in individuals who are male, younger than 57 years, from non-NZE ethnicities, and are more deprived. These findings have important implications for the management of IGT patients and emphasize the need for targeted cancer prevention interventions in this population.

Previous studies have investigated the association between IGT and the risk of cancer in the Swedish general population, and the association between IGT in the Japanese general population [10], the Mauritius general population [9], the Finnish general population [8], the Italian general population [6], and the US general population [7], with varied estimations. A recent meta-analysis showed that individuals with IGT have a higher risk of cancer compared to those with normal glycaemic status (RR: 1.25, 95% CI: 1.02–1.53) [11]. However, few studies have investigated the association between the newly development of T2D and the risk of cancer among individuals with IGT. The present study is the first study to investigate this association and suggests that individuals with IGT who develop T2D have a significantly higher risk of cancer, particularly over the long-term risk (10-year risk), even after accounting for common confounding factors and the competing risk of death from other causes (excluding cancer-related death). Our findings suggest the onset of T2D may play a role in the development of cancer in the population with IGT.

A potential interpretation of the observed increased risk among individuals with IGT who developed T2D could be that T2D and cancer may share common risk factors, such as obesity, socioeconomic deprivation, physical inactivity, and poor diet, which may increase the risk of both conditions [3] [23]. In the current study, BMI and socioeconomic deprivation have been largely ruled out by the tapered matching models. Although ethanol consumption is strongly associated with socioeconomic status and smoking status, which could serve as surrogates for ethanol consumption and were balanced in the analysis, it is important to note that there is a lack of information in the dataset regarding ethanol consumption, which is a major factor linked to other unadjusted factors in carcinogenesis. Data relating to physical activity and diet/nutrition exposure were not available in the current study, which would need further studies to test their impact on the association. Another possible explanation is that chronic hyperglycaemia and insulin resistance, which are significant characteristics among individuals with IGT who develop T2D, may promote cancer development by altering cellular metabolism and promoting the proliferation of cancer cells [24–26]. High levels of glucose and insulin can increase the activity of various growth factors, such as insulin-like growth factor-1 (IGF-1), which can stimulate the growth and survival of cancer cells [27, 28]. In addition, chronic inflammation, observed in the development of T2D, may also play a role in cancer development by promoting DNA damage and impairing the immune response to cancer cells [29, 30].

In the current study, we observed different trends in the adjusted HRs for the 5-year and 10-year cancer risks associated with the onset of T2D. The 5-year risk of cancer showed a decrease over the length of the landmark periods, while the 10-year risk of cancer showed an increase. One possible explanation for these differences is the varying impact of confounding factors over different follow-up durations. Shorter follow-up periods (e.g., 5 years) might not fully capture the long-term biological and environmental influences on cancer development associated with T2D. In contrast, longer follow-up periods (e.g., 10 years) may allow for the cumulative effects of T2D-related metabolic changes, such as chronic hyperglycemia and insulin resistance, which can promote carcinogenesis over time. Moreover, the lack of statistical significance in the 5-year risk compared to the 10-year risk may be due to the relatively shorter follow-up duration, which limits the number of cancer cases observed within this period. This could lead to wider confidence intervals and lower statistical power to detect significant differences. In contrast, the 10-year follow-up period provides a longer observation window, potentially increasing the number of incident cancer cases and thus enhancing the statistical power to detect significant associations. Future research should aim to collect detailed longitudinal data on these lifestyle factors and investigate their long-term interactions with T2D and cancer risk. This could help illustrate the mechanisms underlying the observed temporal differences and improve our understanding of cancer risk dynamics in individuals with T2D.

The main aim of the current study is to understand the association between the onset of T2D and the cancer risk within specific time-windows (5-year and 10-year in the current study), rather than time-to-event. The latter is beyond the scope of this study and could be affected by the recorded event time in the system, potentially introducing information bias. Future studies with accurate records of outcome time are warranted to predict the time to cancer following the development of T2D in the IGT population.

The findings of this study have significant clinical and public health implications. First, our results suggest that individuals with IGT who develop T2D have an increased risk of developing cancer, especially in the first 10 years following the onset of diabetes. Therefore, healthcare providers should be aware of this increased risk and take steps to screen for cancer and provide appropriate counselling to their patients [31]. Additionally, interventions to prevent the onset of T2D may also help to prevent the development of cancer in the IGT population [3]. From a public health perspective, our findings highlight the need for targeted screening strategies for cancer in individuals with IGT, especially those who develop T2D. This is particularly important for individuals from deprived or minority ethnic backgrounds, who were found to be at increased risk of developing cancer in our study. In addition to screening, efforts to promote lifestyle changes, such as smoking cessation and increasing physical activity, may also help to reduce the risk of cancer in this population [32, 33]. By identifying individuals at increased risk of cancer and providing appropriate screening and counselling, healthcare providers and public health officials can take steps to reduce the burden of cancer in this population [34].

Identifying immortal bias is more challenging than confounding effects due to indication. For instance, the association between T2D onset and cancer risk in individuals with IGT exhibited a differential effect when comparing the survival of patients with and without T2D onset. By replacing the original index date with the T2D onset time, the focal population survived from the index date to the T2D onset date, whereas the matched control population could have still been in the early stage of the index date when following up with the onset time as the index date. Although the index date was matched between patients with and without incident T2D, it did not ensure that the time from the index date to the date of T2D onset was comparable between the two groups. Patients who developed T2D were still more prone to having a false survival advantage (being alive at the future cancer diagnosis) because they had to survive until the onset of T2D to be assigned as cases. Hence, without addressing immortal bias by design (e.g., through the use of a landmark analysis), a biased estimate would unavoidably occur.

Our study possesses multiple strengths. Firstly, it is the largest multi-ethnic cohort with IGT studied in NZ and one of the largest globally that investigates the correlation between the onset of T2D and 5- and 10-year cancer risk. These cohorts encompassed all patients from the participating general practices and linked to large, nationally representative databases to prospectively follow patients and record all new cancer cases. The accuracy of clinical recording and diagnoses was validated for outcomes defined by ICD codes, which exhibit high precision. Secondly, the utilization of landmark analysis within 1- to 5-year time frames offered a robust methodology to eliminate immortal bias. Another key strength was the use of an innovative, tapered matching technique to create "quasi-trial" comparison cohorts between patients with IGT with and without an onset of T2Dto compare the risk of 5- and 10-year cancer risk and assess how distinct confounding factors contributed to the risk of cancer. Despite the numerous strengths, limitations exist, including the lack of national representation of the sample and participating general practices in NZ. Lastly, information regarding certain risk factors for cancer such as dietary information, physical activity, and genetic variants was not accessible, and future studies should take these risk factors into account. Future studies with randomised controlled trials or more detailed observational data could further validate our results.

The fundamental challenge of pooling all cancer types lies in the variability of risk factors specific to each type of cancer. While it is well-known that risk factors can vary widely between different cancer types, subdividing the outcomes into exact cancer types would result in very few cases per category, thereby diminishing the statistical power and efficiency of the analysis. The primary aim of this study was to explore the association between the onset of T2D and the overall risk of cancer. By considering the development of T2D as a common risk factor for multiple cancers, we provide a broader perspective that can inform population-level screening strategies and public health interventions. Established findings from our study highlight the importance of implementing preventative measures, such as additional emphasis on smoking cessation programs in primary care, specifically within the IGT population to mitigate the increased cancer risk associated with the onset of T2D. Future studies should consider more detailed causal inference methods to explore specific cancer types once sufficient data are available.

## Conclusion

In conclusion, our study contributes new evidence to the association between the onset of T2D and cancer risk in individuals with IGT, emphasizing the need for increased awareness and targeted interventions to reduce this risk. Clinicians and public health practitioners should consider incorporating lifestyle modifications as part of cancer prevention and management strategies for individuals with IGT. Furthermore, future research should focus on confirming these findings and exploring the underlying mechanisms of the observed associations.

### Supplementary Information

Supplementary material 1.

## Data Availability

The datasets analysed in the current study are not publicly available because of agreements with the primary care organisations and Ministry of Health who provided the data but are available from the corresponding author on reasonable request.

## Abbreviations

T2D: Type 2 diabetes
IGT: Impaired glucose tolerance
NZ: New Zealand
TC: Total cholesterol
SBP: Systolic blood pressure
eGFR: Estimated glomerular filtration rate
NZE: New Zealand European
IGF-1: Insulin-like growth factor-1
CEM: Coarsened exact matching
HR: Hazard ratio
IMD: Index of multiple deprivation
OGTT: An oral glucose tolerance test
DCSS: Diabetes Care Support Service
