# Supplementary material for: Cancer risk following onset of type 2 diabetes in New Zealanders with impaired glucose tolerance over 25 years: a matched prospective cohort study
Source: BMC Cancer. 2024 Jul 24;24:892. doi: 10.1186/s12885-024-12637-4 (PMC11270939; doi:10.1186/s12885-024-12637-4)
Supplement: Supplementary file 1 — Supplementary material 1. [file 12885_2024_12637_MOESM1_ESM.docx]

**Supplemental Material**

**Supplementary Table 1**. Comparison of patients with and without the onset of type 2 diabetes in patients with impaired glucose tolerance

*Categorical variables were presented as count (%); continuous variables were presented as mean (standard deviation).*

|  | **1-year landmark** | | | **2-year landmark** | | | **3-year landmark** | | | **4-year landmark** | | | **5-year landmark** | | |
| --- | --- | --- | --- | --- | --- | --- | --- | --- | --- | --- | --- | --- | --- | --- | --- |
|  | Without T2D onset | With T2D onset | *P*-value | Without T2D onset | With T2D onset | *P*-value | Without T2D onset | With T2D onset | *P*-value | Without T2D onset | With T2D onset | *P*-value | Without T2D onset | With T2D onset | *P*-value |
|  | N=21,648 | N=134 |  | N=21,283 | N=297 |  | N=19,520 | N=454 |  | N=16,681 | N=567 |  | N=13,007 | N=629 |  |
|  | **Unmatched** | | | | | | | | | | | | | | |
| Age, years | 55.1 (12.7) | 55.8 (13.0) | 0.531 | 55.0 (12.7) | 56.6 (12.5) | 0.039 | 54.7 (12.7) | 55.1 (13.4) | 0.555 | 54.7 (12.5) | 54.9 (13.1) | 0.760 | 55.1 (12.3) | 55.1 (12.6) | 0.983 |
| Female Gender, n (%) | 11339 (52.4) | 76 (56.7) | 0.316 | 11133 (52.3) | 160 (53.9) | 0.592 | 10207 (52.3) | 244 (53.7) | 0.540 | 8712 (52.2) | 314 (55.4) | 0.139 | 6773 (52.1) | 338 (53.7) | 0.415 |
| New Zealand European, n (%) | 10334 (47.7) | 54 (40.3) | 0.086 | 10112 (47.5) | 127 (42.8) | 0.103 | 8897 (45.6) | 178 (39.2) | 0.007 | 7725 (46.3) | 219 (38.6) | <0.0001 | 6368 (49.0) | 252 (40.1) | <0.0001 |
| Enrol cohort, n (%) |  |  |  |  |  |  |  |  |  |  |  |  |  |  |  |
| 1994-1998 | 125 (0.6) | 1 (1.5) | <0.0001 | 123 (0.6) | 10 (3.4) | <0.0001 | 119 (0.6) | 11 (2.4) | <0.0001 | 117 (0.7) | 12 (2.1) | <0.0001 | 112 (0.9) | 17 (2.7) | <0.0001 |
| 1999-2003 | 505 (2.3) | 9 (6.7) |  | 495 (2.3) | 23 (7.7) |  | 476 (2.4) | 43 (9.5) |  | 463 (2.8) | 55 (9.7) |  | 456 (3.5) | 70 (11.1) |  |
| 2004-2008 | 1640 (7.6) | 25 (18.7) |  | 1599 (7.5) | 47 (15.8) |  | 1566 (8.02) | 65 (14.3) |  | 1522 (9.1) | 84 (14.8) |  | 1478 (11.4) | 120 (19.1) |  |
| 2009-2013 | 7962 (36.8) | 51 (38.1) |  | 7820 (36.7) | 110 (37.0) |  | 7680 (39.3) | 167 (36.8) |  | 7565 (45.4) | 244 (43.0) |  | 7454 (57.3) | 316 (50.2) |  |
| 2014-2018 | 11416 (52.7) | 47 (35.1) |  | 11246 (52.8) | 107 (36.0) |  | 9679 (49.6) | 168 (37.0) |  | 7014 (42.1) | 172 (30.3) |  | 3507 (27.0) | 106 (16.9) |  |
| IMD group (NZDep13 scale) |  |  |  |  |  |  |  |  |  |  |  |  |  |  |  |
| Least Deprivation: IMD-1 (1 or 2) | 3128 (14.5) | 13 (9.7) | 0.012 | 3071 (14.4) | 26 (8.8) | 0.004 | 2775 (14.2) | 40 (8.8) | 0.004 | 2450 (14.7) | 51 (9.0) | 0.001 | 1834 (14.1) | 49 (7.8) | <0.0001 |
| IMD-2 (3 or 4) | 3797 (17.5) | 12 (9.0) |  | 3743 (17.6) | 40 (13.5) |  | 3293 (16.9) | 69 (15.2) |  | 2856 (17.1) | 88 (15.5) |  | 2259 (17.4) | 98 (15.6) |  |
| IMD-3 (5 or 6) | 2726 (12.6) | 16 (11.9) |  | 2668 (12.5) | 36 (12.1) |  | 2391 (12.3) | 56 (12.3) |  | 1973 (11.8) | 72 (12.7) |  | 1651 (12.7) | 85 (13.5) |  |
| IMD-4 (7 or 8) | 3152 (14.6) | 27 (20.2) |  | 3105 (14.6) | 54 (18.2) |  | 2912 (14.9) | 67 (14.8) |  | 2562 (15.4) | 84 (14.8) |  | 2073 (15.9) | 104 (16.5) |  |
| Most Deprivation: IMD-5 (9 or 10) | 8845 (40.9) | 66 (49.3) |  | 8696 (40.9) | 141 (47.5) |  | 8149 (41.8) | 222 (48.9) |  | 6840 (41.0) | 272 (48.0) |  | 5190 (39.9) | 293 (46.6) |  |
| Smoking status, n (%) |  |  |  |  |  |  |  |  |  |  |  |  |  |  |  |
| Never smoking | 12939 (60.0) | 74 (55.2) | 0.504 | 12738 (59.9) | 159 (53.5) | 0.075 | 11688 (59.9) | 248 (54.6) | 0.071 | 10040 (60.2) | 318 (56.1) | 0.143 | 7908 (60.8) | 354 (56.3) | 0.057 |
| Ex-smoker | 5561 (25.7) | 40 (29.9) |  | 5447 (25.6) | 91 (30.6) |  | 4939 (25.3) | 127 (28.0) |  | 4201 (25.2) | 156 (27.5) |  | 3247 (25.0) | 169 (26.9) |  |
| Current Smoker | 3148 (14.5) | 20 (14.9) |  | 3098 (14.6) | 47 (15.8) |  | 2893 (14.8) | 79 (17.4) |  | 2440 (14.6) | 93 (16.4) |  | 1852 (14.2) | 106 (16.9) |  |
| Body mass index, kg/m^2^ | 31.2 (6.4) | 33.8 (7.4) | <0.0001 | 31.3 (6.4) | 33.7 (7.1) | <0.0001 | 31.3 (6.4) | 33.9 (7.0) | <0.0001 | 31.2 (6.3) | 33.6 (6.8) | <0.0001 | 31.1 (6.2) | 33.8 (6.8) | <0.0001 |
| Systolic blood pressure, mmHg | 131 (16) | 131 (17) | 0.968 | 131 (16) | 132 (17) | 0.183 | 131 (16) | 132 (17) | 0.107 | 131 (16) | 133 (18) | 0.001 | 131 (16) | 133 (18) | 0.002 |
| Diastolic blood pressure, mmHg | 79 (10) | 79 (10) | 0.799 | 79 (10) | 81 (11) | 0.037 | 79 (10) | 81 (11) | 0.009 | 80 (10) | 81 (11) | <0.0001 | 80 (10) | 81 (11) | 0.003 |
| HbA1c, mmol/mol | 42.2 (3.2) | 43.2 (3.7) | 0.001 | 42.2 (3.2) | 43.8 (3.8) | <0.0001 | 42.3 (3.2) | 44.3 (4.2) | <0.0001 | 42.5 (3.1) | 44.7 (4.0) | <0.0001 | 42.6 (3.1) | 44.8 (4.1) | <0.0001 |
| Total cholesterol, mmol/L | 5.1 (1.0) | 4.8 (1.0) | <0.0001 | 5.1 (1.0) | 4.8 (1.0) | <0.0001 | 5.1 (1.0) | 4.9 (1.0) | <0.0001 | 5.1 (1.0) | 4.9 (1.0) | <0.0001 | 5.1 (1.0) | 4.9 (1.0) | <0.0001 |
| Triglyceride, mmol/L | 1.7 (0.7) | 1.6 (0.7) | 0.140 | 1.7 (0.8) | 1.7 (0.8) | 0.351 | 1.7 (0.8) | 1.8 (0.8) | 0.004 | 1.7 (0.8) | 1.8 (0.8) | 0.002 | 1.6 (0.8) | 1.8 (0.8) | 0.001 |
| Low-density lipoprotein cholesterol, mmol/L | 2.9 (0.7) | 2.6 (0.7) | <0.0001 | 2.9 (0.7) | 2.5 (0.7) | <0.0001 | 2.9 (0.7) | 2.7 (0.7) | <0.0001 | 2.8 (0.7) | 2.7 (0.8) | <0.0001 | 2.9 (0.7) | 2.7 (0.7) | <0.0001 |
| High-density lipoprotein cholesterol, mmol/L | 1.3 (0.4) | 1.3 (0.4) | 0.556 | 1.3 (0.4) | 1.3 (0.4) | 0.339 | 1.3 (0.4) | 1.3 (0.3) | 0.008 | 1.3 (0.4) | 1.2 (0.3) | <0.0001 | 1.3 (0.4) | 1.2 (0.3) | <0.0001 |
| estimated Glomerular filtration rate<90 ml/min/1.73 m^2^ | 6968 (32.2) | 49 (36.6) | 0.007 | 6078 (32.3) | 106 (35.7) | <0.0001 | 6491 (33.3) | 171 (37.7) | <0.0001 | 5835 (35.0) | 227 (40.0) | <0.0001 | 3672 (28.2) | 207 (32.9) | 0.009 |
| Antihypertensive treatment, n (%) | 2195 (10.1) | 36 (26.9) | <0.0001 | 2150 (10.1) | 82 (27.6) | <0.0001 | 2088 (10.7) | 131 (28.9) | <0.0001 | 2010 (12.2) | 171 (30.2) | <0.0001 | 1902 (14.6) | 224 (35.6) | <0.0001 |
| Statin treatment, n (%) | 1836 (8.5) | 34 (25.4) | <0.0001 | 1806 (8.5) | 71 (23.9) | <0.0001 | 1755 (9.0) | 115 (25.3) | <0.0001 | 1689 (10.1) | 153 (27.0) | <0.0001 | 1605 (12.3) | 207 (32.9) | <0.0001 |
| Antiplatelet or anticoagulant treatment, n (%) | 61 (0.3) | 2 (1.5) | 0.009 | 61 (0.3) | 3 (1.0) | 0.023 | 60 (0.3) | 7 (1.5) | <0.0001 | 54 (0.3) | 7 (1.2) | <0.0001 | 52 (0.4) | 9 (1.4) | <0.0001 |
|  | **Coarsened and exact matched** | | | | | | | | | | | | | | |
| N | n=1,435 | N=112 |  | n=2,872 | n=254 |  | n=3,818 | n=385 |  | n=3,922 | n=477 |  | n=3,336 | n=511 |  |
| Age, years | 52.6 (13.9) | 56.3 (13.1) | 0.007 | 53.2 (13.5) | 57.0 (12.5) | <0.0001 | 52.2 (13.0) | 55.2 (13.6) | <0.0001 | 52.6 (12.9) | 54.9 (13.3) | <0.0001 | 52.2 (12.2) | 54.9 (12.9) | <0.0001 |
| Female Gender, n (%) | 852 (59.4) | 63 (56.3) | 0.517 | 1440 (50.1) | 133 (52.4) | 0.497 | 1937 (50.7) | 198 (51.4) | 0.795 | 2102 (53.6) | 259 (54.3) | 0.771 | 1729 (51.8) | 273 (53.4) | 0.501 |
| New Zealand European, n (%) | 356 (24.8) | 39 (34.8) | 0.019 | 821 (28.6) | 102 (40.2) | <0.0001 | 984 (25.8) | 143 (37.1) | <0.0001 | 1171 (29.9) | 183 (38.4) | <0.0001 | 970 (29.1) | 202 (39.5) | <0.0001 |
| Enrol cohort, n (%) |  |  |  |  |  |  |  |  |  |  |  |  |  |  |  |
| 1994-1998 | 4 (0.3) | 2 (1.8) | <0.0001 | 9 (0.3) | 10 (3.9) | <0.0001 | 8 (0.2) | 10 (2.6) | <0.0001 | 14 (0.4) | 9 (1.9) | <0.0001 | 15 (0.5) | 13 (2.5) | <0.0001 |
| 1999-2003 | 26 (1.8) | 4 (3.6) |  | 53 (1.9) | 15 (5.9) |  | 81 (2.1) | 34 (8.8) |  | 109 (2.8) | 45 (9.4) |  | 100 (3.0) | 56 (11.0) |  |
| 2004-2008 | 73 (5.1) | 20 (17.9) |  | 167 (5.8) | 37 (14.6) |  | 211 (5.5) | 49 (12.7) |  | 283 (7.2) | 63 (13.2) |  | 293 (8.8) | 81 (15.9) |  |
| 2009-2013 | 672 (46.8) | 44 (39.3) |  | 1274 (44.4) | 95 (37.4) |  | 1726 (45.2) | 144 (37.4) |  | 2190 (55.8) | 209 (43.8) |  | 2499 (74.9) | 274 (53.6) |  |
| 2014-2018 | 660 (46.0) | 42 (37.5) |  | 1369 (47.7) | 97 (38.2) |  | 1792 (46.9) | 148 (38.4) |  | 1326 (33.8) | 151 (31.7) |  | 429 (18.9) | 87 (17.0) |  |
| IMD group (NZDep13 scale) |  |  |  |  |  |  |  |  |  |  |  |  |  |  |  |
| Least Deprivation: IMD-1 (1 or 2) | 97 (6.8) | 10 (8.9) | 0.046 | 215 (7.5) | 18 (7.1) | <0.0001 | 298 (7.8) | 30 (7.8) | <0.0001 | 391 (10.0) | 41 (8.6) | 0.030 | 318 (9.5) | 39 (7.6) | 0.028 |
| IMD-2 (3 or 4) | 122 (8.5) | 11 (9.8) |  | 273 (9.5) | 37 (14.6) |  | 401 (10.5) | 60 (15.6) |  | 495 (12.6) | 75 (15.7) |  | 470 (14.1) | 80 (15.7) |  |
| IMD-3 (5 or 6) | 91 (6.3) | 12 (10.7) |  | 196 (6.8) | 29 (11.4) |  | 287 (7.5) | 46 (12.0) |  | 348 (8.9) | 58 (12.2) |  | 308 (9.2) | 65 (12.7) |  |
| IMD-4 (7 or 8) | 219 (15.3) | 24 (21.4) |  | 443 (15.4) | 48 (18.9) |  | 585 (15.3) | 58 (15.1) |  | 590 (15.0) | 70 (14.7) |  | 493 (14.8) | 84 (16.4) |  |
| Most Deprivation: IMD-5 (9 or 10) | 906 (63.1) | 55 (49.1) |  | 1745 (60.8) | 122 (48.0) |  | 2247 (58.9) | 191 (49.6) |  | 2098 (53.5) | 233 (48.9) |  | 1747 (52.4) | 243 (47.6) |  |
| Smoking status, n (%) |  |  |  |  |  |  |  |  |  |  |  |  |  |  |  |
| Never smoking | 751 (52.3) | 59 (52.7) | 0.488 | 1477 (51.4) | 136 (53.5) | 0.465 | 2112 (55.3) | 215 (55.8) | 0.932 | 2359 (60.2) | 276 (57.9) | 0.415 | 1990 (59.7) | 289 (56.6) | 0.303 |
| Ex-smoker | 394 (27.5) | 35 (31.3) |  | 828 (28.8) | 76 (29.9) |  | 992 (26.0) | 101 (26.2) |  | 897 (22.9) | 122 (25.6) |  | 757 (22.7) | 131 (25.6) |  |
| Current Smoker | 299 (20.2) | 18 (16.1) |  | 567 (19.7) | 42 (16.5) |  | 714 (18.7) | 69 (17.9) |  | 666 (17.0) | 79 (16.6) |  | 589 (17.7) | 91 (17.8) |  |
| Body mass index, kg/m^2^ | 34.0 (6.6) | 34.1 (7.5) | 0.915 | 33.5 (6.5) | 33.7 (7.2) | 0.666 | 33.6 (6.4) | 33.9 (7.1) | 0.378 | 33.2 (6.5) | 33.6 (6.8) | 0.260 | 33.0 (6.5) | 33.9 (6.9) | 0.006 |
| Systolic blood pressure, mmHg | 131 (17) | 131 (16) | 0.951 | 134 (16) | 132 (17) | 0.238 | 132 (16) | 132 (16) | 0.957 | 132 (16) | 134 (18) | 0.102 | 132 (16) | 133 (17) | 0.024 |
| Diastolic blood pressure, mmHg | 80 (10) | 79 (10) | 0.275 | 81 (10) | 81 (11) | 0.529 | 81 (10) | 81 (11) | 0.815 | 81 (10) | 82 (11) | 0.312 | 81 (10) | 82 (11) | 0.429 |
| HbA1c, mmol/mol | 43.3 (3.6) | 43.6 (3.5) | 0.380 | 43.4 (2.7) | 44.1 (3.6) | <0.0001 | 43.4 (2.7) | 44.6 (4.0) | <0.0001 | 43.5 (2.7) | 44.9 (3.8) | <0.0001 | 43.5 (2.7) | 45.0 (3.8) | <0.0001 |
| Total cholesterol, mmol/L | 5.1 (0.9) | 4.8 (1.0) | 0.001 | 5.2 (0.9) | 4.9 (1.0) | <0.0001 | 5.2 (0.9) | 4.9 (1.0) | <0.0001 | 5.2 (0.9) | 5.0 (1.0) | <0.0001 | 5.1 (0.9) | 5.0 (1.0) | <0.0001 |
| Triglyceride, mmol/L | 1.7 (0.8) | 1.6 (0.7) | 0.077 | 1.8 (0.8) | 1.7 (0.8) | 0.640 | 1.8 (0.8) | 1.8 (0.8) | 0.391 | 1.7 (0.8) | 1.8 (0.7) | 0.274 | 1.7 (0.8) | 1.8 (0.7) | 0.204 |
| Low-density lipoprotein cholesterol, mmol/L | 2.9 (0.7) | 2.7 (0.7) | 0.001 | 2.9 (0.7) | 2.7 (0.7) | <0.0001 | 2.9 (0.7) | 2.7 (0.7) | <0.0001 | 3.0 (0.7) | 2.7 (0.7) | <0.0001 | 3.0 (0.7) | 2.7 (0.7) | <0.0001 |
| High-density lipoprotein cholesterol, mmol/L | 1.2 (0.3) | 1.3 (0.4) | 0.014 | 1.3 (0.4) | 1.3 (0.5) | 0.357 | 1.2 (0.4) | 1.3 (0.3) | 0.739 | 1.3 (0.4) | 1.2 (0.3) | 0.417 | 1.3 (0.4) | 1.2 (0.3) | 0.038 |
| estimated Glomerular filtration rate<90 ml/min/1.73 m^2^ | 505 (35.2) | 41 (36.6) | 0.001 | 1060 (36.9) | 89 (35.0) | 0.010 | 1440 (37.7) | 140 (36.4) | 0.077 | 1604 (40.9) | 188 (39.4) | 0.067 | 669 (20.1) | 154 (30.1) | <0.0001 |
| Antihypertensive treatment, n (%) | 104 (7.3) | 27 (24.1) | <0.0001 | 240 (8.4) | 58 (22.8) | <0.0001 | 324 (8.5) | 95 (24.7) | <0.0001 | 405 (10.3) | 122 (25.6) | <0.0001 | 426 (12.8) | 163 (31.9) | <0.0001 |
| Statin treatment, n (%) | 86 (6.0) | 26 (23.2) | <0.0001 | 196 (6.8) | 54 (21.3) | <0.0001 | 267 (7.0) | 84 (21.8) | <0.0001 | 334 (0.5) | 110 (23.1) | <0.0001 | 365 (10.9) | 151 (30.0) | <0.0001 |
| Antiplatelet or anticoagulant treatment, n (%) | 8 (0.6) | 2 (1.8) | 0.118 | 9 (0.3) | 3 (1.2) | 0.032 | 13 (0.3) | 4 (1.0) | 0.040 | 13 (0.3) | 3 (0.6) | 0.308 | 12 (0.4) | 4 (0.8) | 0.166 |

**Supplementary Figure 1**. Graphical representation of landmark analysis.


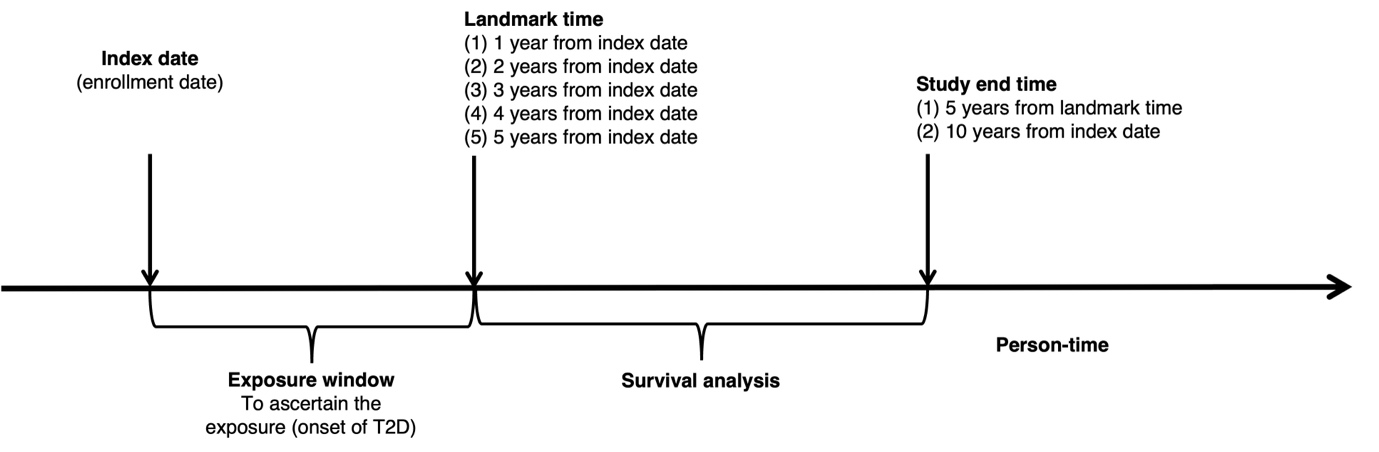


**Supplementary Figure 2**. Workflow charts for matching process (1-year landmark analysis)


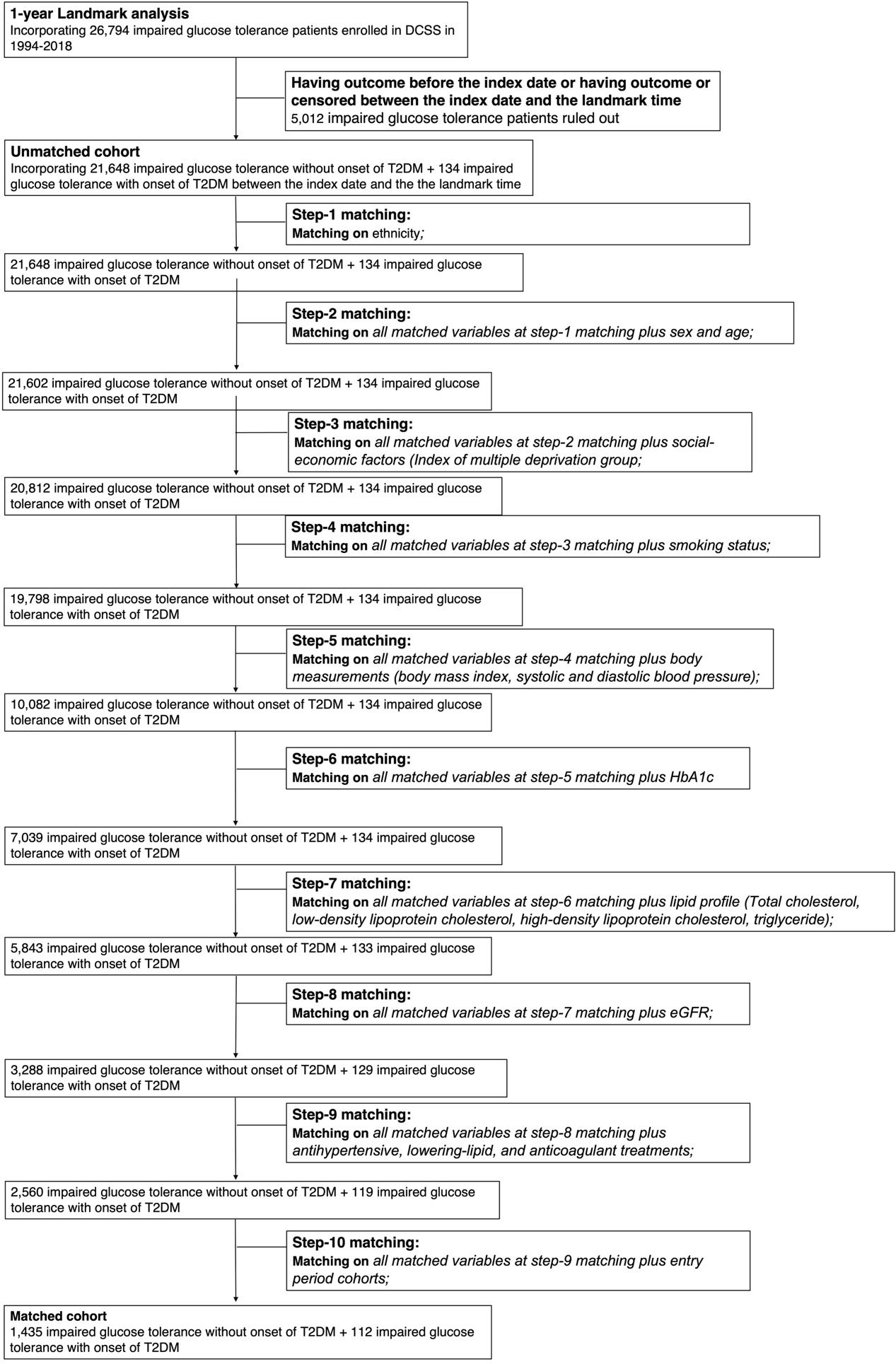


**Supplementary Figure 3**. Workflow charts for matching process (2-year landmark analysis)


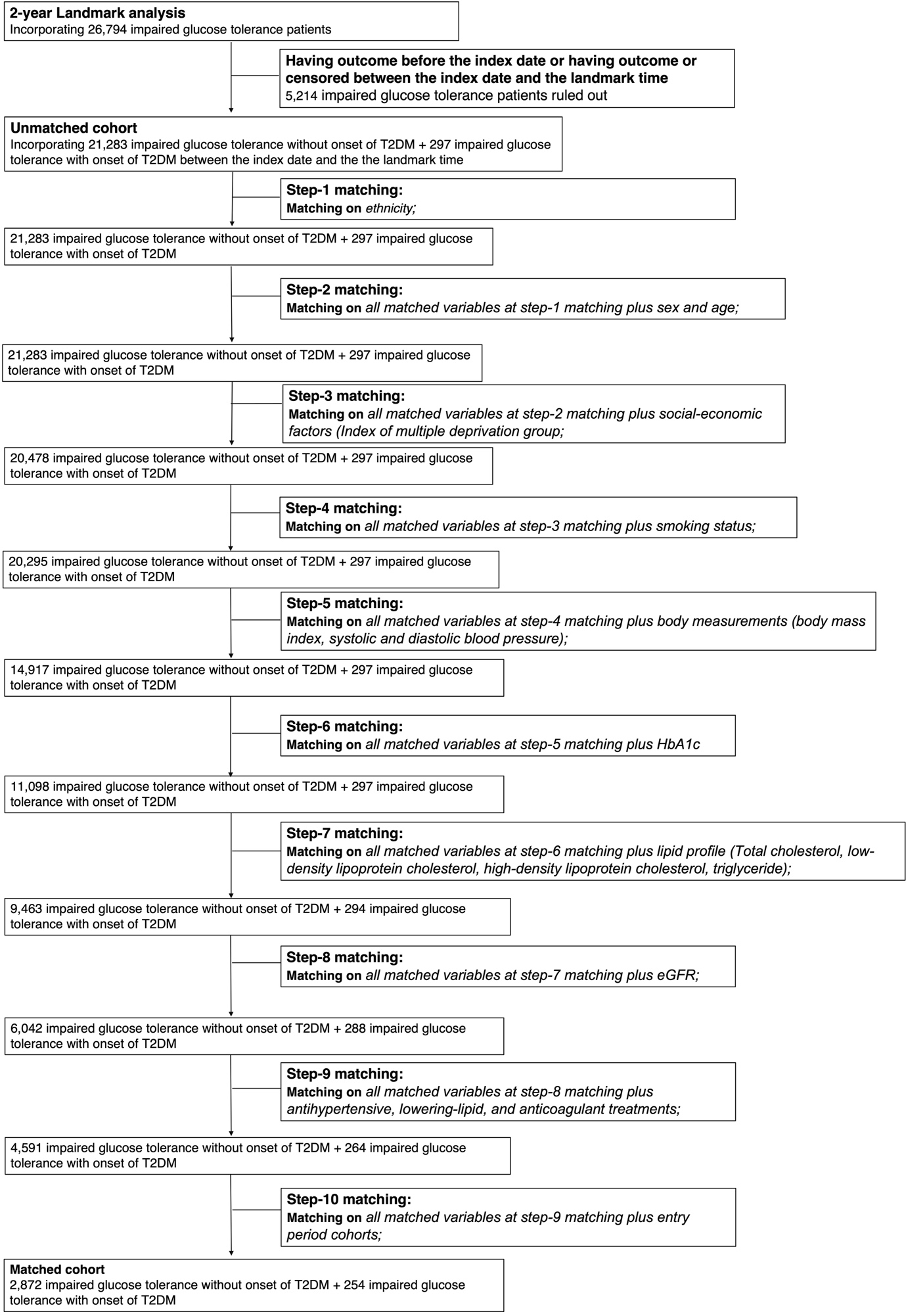


**Supplementary Figure 4**. Workflow charts for matching process (3-year landmark analysis)


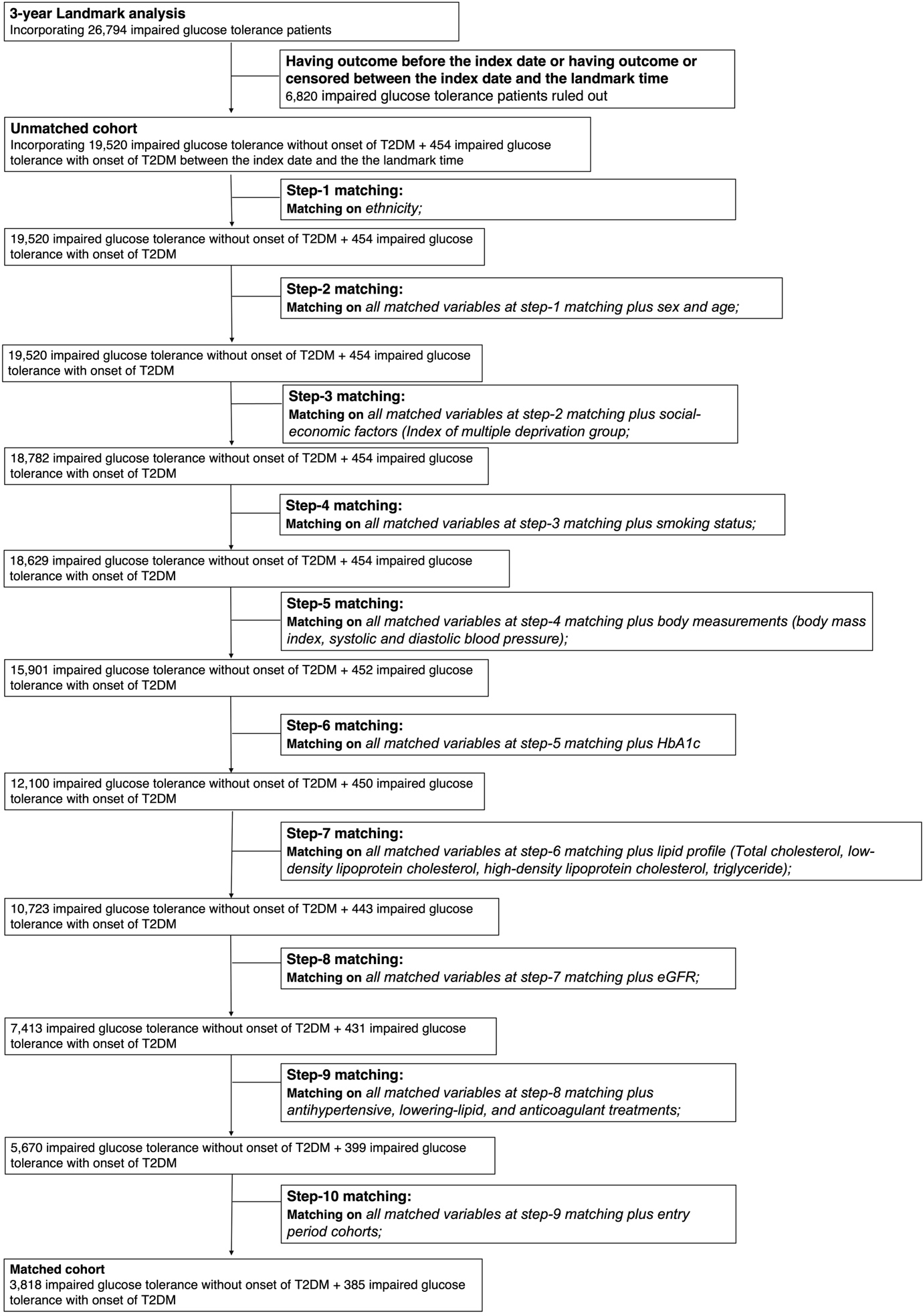


**Supplementary Figure 5**. Workflow charts for matching process (4-year landmark analysis)


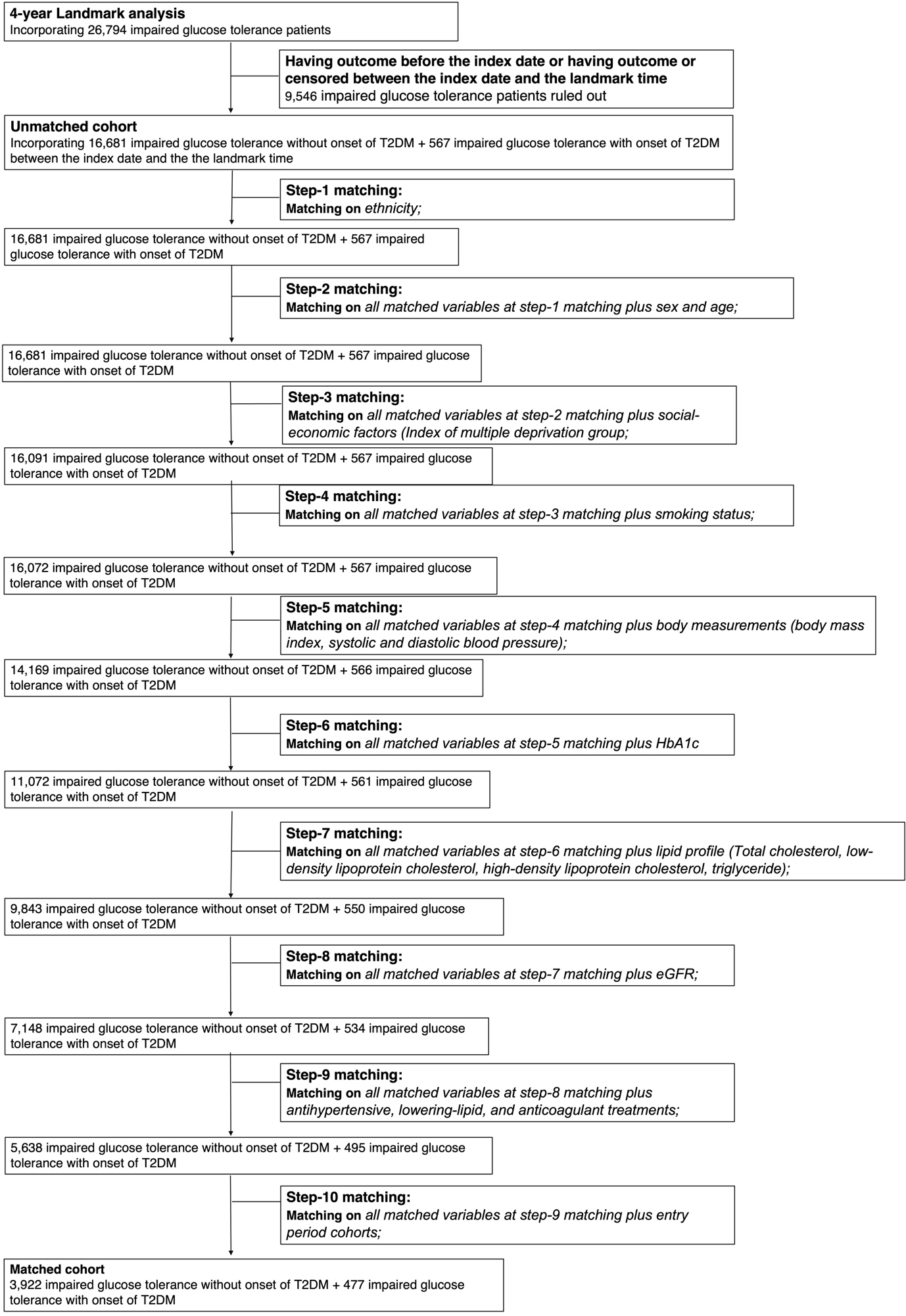


**Supplementary Figure 6**. Workflow charts for matching process (5-year landmark analysis)


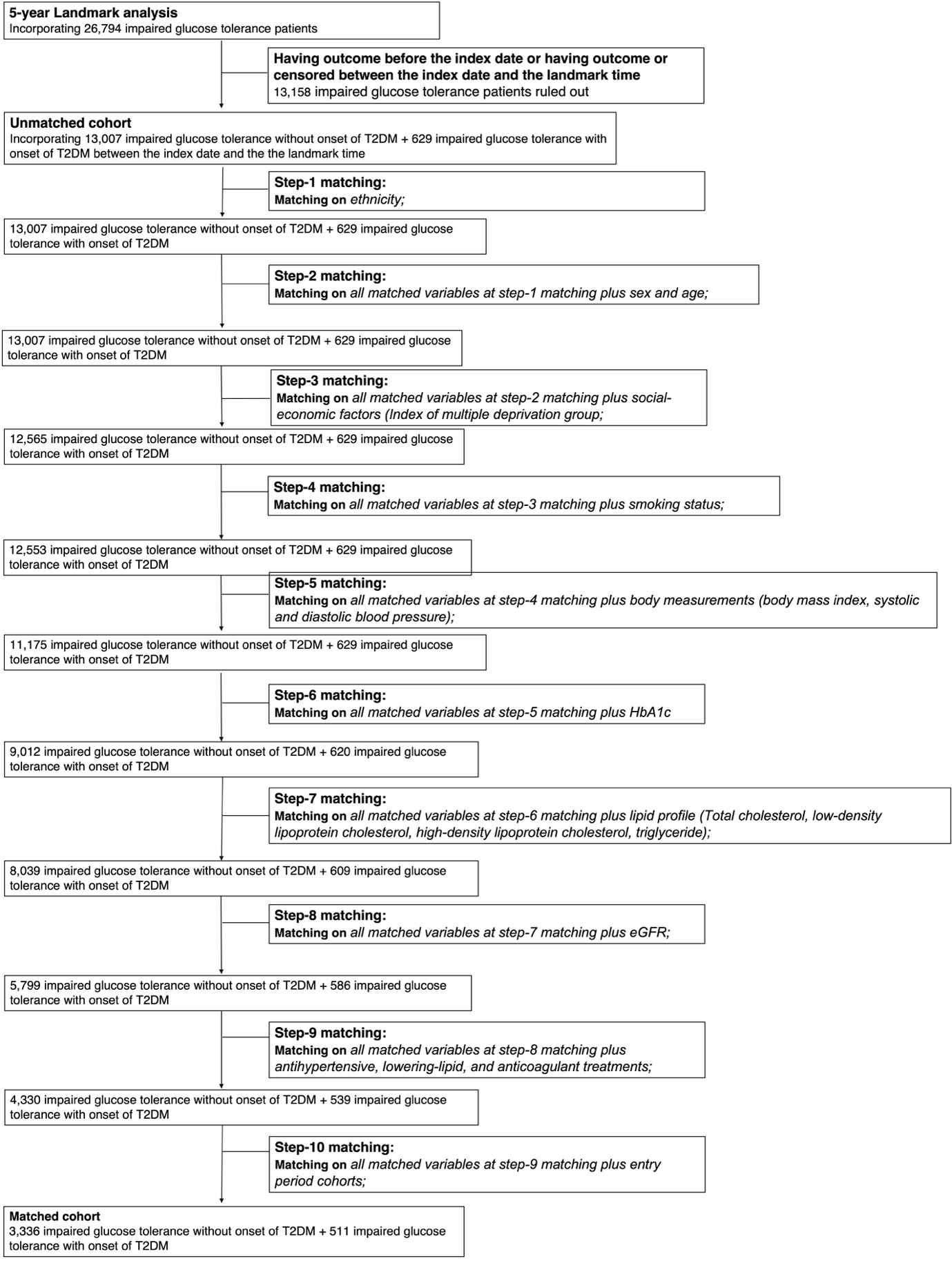


**Supplementary Figure 7**. Adjusted risk ratios for 5-year risk of cancer between patients with impaired glucose tolerance with and without the onset of type 2 diabetes

*Model (i) weighted for ethnicity; model (ii) weighted for all adjusted variables in model (i) plus age and sex; model (iii) weighted for all adjusted variables in model (ii) plus IMD group; model (iv) weighted for all adjusted variables in model (iii) plus smoking status; model (v) weighted for all adjusted variables in model (iv) plus body measurements (body mass index, systolic and diastolic blood pressure); model (vi) weighted for all adjusted variables in model (v) plus baseline HbA1c; model (vii) weighted for all adjusted variables in model (vi) plus baseline lipid profile (total cholesterol, low-density lipoprotein cholesterol, high-density lipoprotein cholesterol, and triglyceride); model (viii) weighted for all adjusted variables in model (vii) plus eGFR; model (ix) weighted for all adjusted variables in model (viii) plus antihypertensive, lowering lipid and anticoagulant treatment; model (x) weighted for all adjusted variables in model (ix) plus entry cohorts; The log-scale for Y-axis (risk ratio) was applied.*

**Supplementary Figure 8**. Adjusted risk ratios for 10-year risk of cancer between people with impaired glucose tolerance with and without the onset of type 2 diabetes

*Model (i) weighted for ethnicity; model (ii) weighted for all adjusted variables in model (i) plus age and sex; model (iii) weighted for all adjusted variables in model (ii) plus IMD group; model (iv) weighted for all adjusted variables in model (iii) plus smoking status; model (v) weighted for all adjusted variables in model (iv) plus body measurements (body mass index, systolic and diastolic blood pressure); model (vi) weighted for all adjusted variables in model (v) plus baseline HbA1c; model (vii) weighted for all adjusted variables in model (vi) plus baseline lipid profile (total cholesterol, low-density lipoprotein cholesterol, high-density lipoprotein cholesterol, and triglyceride); model (viii) weighted for all adjusted variables in model (vii) plus eGFR; model (ix) weighted for all adjusted variables in model (viii) plus antihypertensive, lowering lipid and anticoagulant treatment; model (x) weighted for all adjusted variables in model (ix) plus entry cohorts; The log-scale for Y-axis (risk ratio) was applied.*
